# Fisetin Confers Cardioprotection against Myocardial Ischemia Reperfusion Injury by Suppressing Mitochondrial Oxidative Stress and Mitochondrial Dysfunction and Inhibiting Glycogen Synthase Kinase 3*β* Activity

**DOI:** 10.1155/2018/9173436

**Published:** 2018-02-25

**Authors:** Karthi Shanmugam, Sriram Ravindran, Gino A. Kurian, Mohanraj Rajesh

**Affiliations:** ^1^SASTRA Deemed University, Vascular Biology Laboratory, School of Chemical and Biotechnology, Thanjavur, Tamil Nadu 613401, India; ^2^Department of Pharmacology and Therapeutics, College of Medicine and Health Sciences, UAE University, Al Ain 17666, UAE

## Abstract

Acute myocardial infarction (AMI) is the leading cause of morbidity and mortality worldwide. Timely reperfusion is considered an optimal treatment for AMI. Paradoxically, the procedure of reperfusion can itself cause myocardial tissue injury. Therefore, a strategy to minimize the reperfusion-induced myocardial tissue injury is vital for salvaging the healthy myocardium. Herein, we investigated the cardioprotective effects of fisetin, a natural flavonoid, against ischemia/reperfusion (I/R) injury (IRI) using a Langendorff isolated heart perfusion system. I/R produced significant myocardial tissue injury, which was characterized by elevated levels of lactate dehydrogenase and creatine kinase in the perfusate and decreased indices of hemodynamic parameters. Furthermore, I/R resulted in elevated oxidative stress, uncoupling of the mitochondrial electron transport chain, increased mitochondrial swelling, a decrease of the mitochondrial membrane potential, and induction of apoptosis. Moreover, IRI was associated with a loss of the mitochondrial structure and decreased mitochondrial biogenesis. However, when the animals were pretreated with fisetin, it significantly attenuated the I/R-induced myocardial tissue injury, blunted the oxidative stress, and restored the structure and function of mitochondria. Mechanistically, the fisetin effects were found to be mediated via inhibition of glycogen synthase kinase 3*β* (GSK3*β*), which was confirmed by a biochemical assay and molecular docking studies.

## 1. Introduction

Ischemic heart disease is one of the leading causes of morbidity and mortality worldwide, while effective therapy to limit the spectrum of abnormalities often leads to arrhythmias, myocardial stunning, and necrosis, and these pathological changes are collectively referred to as reperfusion injury (RI) [1, 2]. Clinically, predicting the onset of RI is challenging, because of the lack of definitive biomarkers. This problem is confounded by the lack of clear understanding of the pathomechanisms involved in RI [3]. Currently, mitochondrial dysfunction, oxidative stress, perturbed cardiomyocyte Ca^2+^ homeostasis, inflammation, and apoptosis cascades are recognized as the key drivers for RI-induced myocardial tissue damage [4, 5]. Therefore, targeting these pathways could be beneficial for preventing RI and could aid in the functional recovery of the myocardium.

Epidemiological studies have indicated that regular consumption of fruits and vegetables containing flavonoids and polyphenols is associated with a reduced risk for the development of cardiovascular diseases, inflammatory diseases, neurodegenerative diseases, and cancer [6]. The underlying mechanisms of several cardioprotective procedures such as ischemic preconditioning for IRI have been linked to the inhibition of GSK3*β*, which provides cardioprotection by modulating mitochondrial ATP-sensitive K^+^ channel, the mammalian target of rapamycin (mTOR) signaling pathway, and autophagy [7–9]. Since downstream signaling of GSK3*β* converges in mitochondria, along with its translocation by ischemic stimuli, studying the action of natural compounds on reversing mitochondrial dysfunction, the key player in the pathology of I/R, by modulating GSK3*β* will provide the impetus for the development of small molecules as selective GSK3*β* inhibitor [8, 10]. Fisetin is a natural flavonoid found in several fruits and vegetables [11]. The compound has been reported to elicit antioxidant and anti-inflammatory effects, retard the development of atherosclerosis, and exhibit neuroprotective properties in several preclinical studies [11]. In this study, we investigated the cardioprotective effects of fisetin against IRI using a Langendorff isolated heart perfusion system. In addition, in in silico and molecular docking studies, using the crystal structure of GSK3*β*, a library of polyphenolic compounds was screened by energy-optimized pharmacophore-based virtual screening to identify a molecule with the best-suited structural orientation for GSK3*β* inhibition. Since fisetin emerged as the prime candidate for a GSK3*β* inhibitor, its cardioprotective activity was validated using an isolated rat heart model of IRI.

## 2. Materials and Methods

### 2.1. Animals and Chemicals

All animal experimental procedures were conducted according to the guidelines of the Committee for the Purpose of Control and Supervision of Experiments on Animals (CPCSEA), Government of India. A prior approval for the conduct of experiments was obtained from the Institutional Animal Ethical Committee (IAEC) at SASTRA Deemed University, Thanjavur, India. Male Wistar rats (250–300 g) used in the study were inbred at the central animal facility, SASTRA University. All the animals were housed in ventilated polycarbonate cages and provided access to food and water ad libitum. Unless specified, all fine chemicals were procured from Sigma-Aldrich (St. Louis, MO, USA).

### 2.2. Isolated Rat Heart Model of Ischemia Reperfusion Injury

Isolated mammalian heart model according to Langendorff was used for establishment of myocardial IRI [12]. The ex vivo method involved anesthetization of a rat (ketamine 80 mg/kg + xylazine 20 mg/kg) followed by the excision of heart and perfusion with Krebs-Henseleit buffer (118.0 mM NaCl, 4.7 mM KCl, 1.9 mM CaCl_2_, 1.2 mM MgSO_4_, 25.0 mM NaHCO_3_, 1.2 mM KH_2_PO_4_, and 10.1 mM glucose, pH 7.4), maintained at 37°C with continuous oxygenation (95% O_2_+ 5% CO_2_). The heart was stabilized for 20 min on the perfusion system (ADInstruments, Bella Vista, New South Wales, Australia) by maintaining a constant perfusate pressure of 70 mmHg. Hemodynamic changes were monitored using a pressure transducer connected to a latex balloon placed in the left ventricle. Electrical recordings were continuously made using a PowerLab data acquisition system (ADInstruments) and analyzed using the LabChart Pro 8 software (ADInstruments) [12].

The experimental groups included sham, fisetin + sham, I/R alone, and fisetin pretreatment, followed by I/R (fisetin + I/R). Fisetin (20 mg/kg; TOCRIS Bioscience, Bristol, UK) was injected intraperitoneally 1 h before the induction of ischemia. We performed pilot experiments to ascertain a suitable dosage regimen of fisetin, and our observations revealed that administration of fisetin at (20 mg/kg) consistently provided the optimal cardioprotection. Therefore, we adopted this dose for further experiments.

A typical I/R protocol consisted of 30 min of ischemia induced by stopping the buffer flow, followed by 60 min of reperfusion induced by resuming the flow. Throughout the duration of the experiment, hemodynamic parameters were continuously monitored and the perfusate was collected at the end of reperfusion for biochemical analysis. At the end of the experiment, the hearts were immediately frozen in liquid nitrogen and stored at −80°C until further analysis.

### 2.3. Functional and Morphological Assessment of Cardiac Injury

Cardiac injury was assessed by measuring the levels of lactate dehydrogenase (LDH) and creatine kinase (CK) released into the perfusate after ischemic injury. LDH was estimated spectrophotometrically at a wavelength of 340 nm based on the conversion of lactate to pyruvate and expressed as NADH oxidized/min/mg protein [13]. CK was estimated based on the amount of inorganic phosphate formed from ATP, with 1-amino-2-naphthol-4-sulphonic acid (ANSA) reagent and measured at 640 nm [13]. Heart sections were stained using triphenyl tetrazolium chloride (TTC) to calculate the percentage of the infarcted area. Measurement of the infarct size was performed as described previously [14]. Briefly, heart sections were incubated in 1.5% TTC prepared in PBS for 10 min at 37°C. Images were acquired using a zoom stereomicroscope (NIKON-SMZ1270) equipped with a high-definition CCD camera (NIKON-DS-Fi2), and the NIS Elements documentation software. The ImageJ software (NIH-USA) was used for the measurement of the infarcted area [14].

### 2.4. Isolation of Subcellular Organelles (Mitochondria, Lysosomes, and Microsomes)

Cardiac mitochondria were isolated by differential centrifugation as described previously [15], and microsomes and lysosomes were isolated using sucrose density gradients as described by Graham [16]. Briefly, a 10% heart homogenate was prepared in isolation buffer (220 mM mannitol, 70 mM sucrose, 5 mM MOPS, 2 mM EDTA, and 0.2% BSA, pH 7.4) and centrifuged at 500 ×g for 10 min at 4°C, to pellet the nuclear fraction. The resulting supernatant was centrifuged at 12,000 ×g for 10 min at 4°C to obtain a crude mitochondrial fraction consisting of light and heavy mitochondria as well as lysosomes. In the subsequent step, the supernatant, consisting of microsomes, was ultracentrifuged (Beckman Coulter, Indianapolis, IN, USA) at 100,000 ×g for 40 min at 4°C to collect microsomes, and the supernatant was used as the cytosolic fraction for further analysis. The microsomes were resuspended in storage buffer (0.25 M sucrose, 1 mM EDTA, and 10 mM HEPES adjusted to pH 7.4).

The crude mitochondrial pellet was subjected to sucrose density gradient separation as reported earlier [17]. Briefly, a sucrose density gradient (1.25, 1.22, 1.19, 1.15, 1.11, and 1.09 g/cm^3^) was prepared in PBS (pH 7.4) and layered in a 5 mL centrifuge tube. The crude mitochondrial pellet was resuspended in PBS, then layered on top of the density gradient, and centrifuged in a swingout bucket rotor at 100,000 ×g for 3 h at 4°C using the lowest acceleration and deceleration speeds. Based on their densities, lysosomes (1.12 g/cm^3^) and mitochondria (1.18 g/cm^3^) were separated into two fractions, which were collected and stored in storage buffer (0.25 M sucrose, 1 mM EDTA, and 10 mM HEPES adjusted to pH 7.4). All the subcellular fractions separated were estimated for their protein content using the Bradford assay reagent (Bio-Rad, Hercules, CA, USA).

### 2.5. Estimation of Lipid Peroxide, Antioxidant, and Antioxidant Enzyme Activities in Subcellular Compartments

The cytosolic and subcellular fractions isolated as described above were used for the assessment of oxidative stress markers. A thiobarbituric acid reactive species (TBARS) assay based on the formation of malondialdehyde was performed to quantify the amount of lipid peroxidation by using the method of Ohkawa et al. [18]. Briefly, 100 *μ*L of a sample was added to the reaction mixture (0.8% thiobarbituric acid and 15% trichloroacetic acid, adjusted to pH 3.5), and the mixture was incubated for 1 h in a water bath maintained at 75°C. After cooling, a butanol-pyridine mixture (15 : 1) was added to extract the pink-colored complex, and the absorbance of the organic layer was read at 532 nm. Malondialdehyde was used as a standard in the assay [12, 13, 15].

The antioxidant status of the myocardium was evaluated based on the GSH level using Ellman's reagent (5,5′-dithiobis-2-nitro-benzoic acid). GSH concentration was determined according to the method of Sedlak and Lindsay [19]. In brief, phosphate buffer (pH 8), 5% trichloroacetic acid, and Ellman's reagent were added to a protein sample to observe the color development due to formation of thionitrobenzoate. The absorbance was measured at 412 nm, and the values were expressed as *μ*M/*μ*g of protein using GSH as a standard [12, 13, 15].

The superoxide dismutase (SOD) activity was determined in cardiac tissues as reported [12, 13, 15]. The assay was based on the ability of SOD, present in the samples, to inhibit the autoxidation of pyrogallol. Briefly, 50 *μ*L of a sample was mixed with buffer (45 mM Tris, 1 mM EDTA adjusted to pH 7.4), and 2.5 mM pyrogallol was added to initiate the reaction. The autoxidation of pyrogallol was kinetically monitored at 420 nm for 5 min, and the SOD activity was calculated based on the ability of 1 unit of SOD to inhibit autoxidation of pyrogallol by 50%.

The catalase activity was determined as reported [12, 13, 15]. Essentially, the reaction buffer (0.1 M sodium phosphate buffer, pH 7.2, 4 mM H_2_O_2_, and 5 N H_2_SO_4_) was added to 100 *μ*L of a sample, and the reaction was initiated by the addition of 0.005 M KMnO_4_. The rate of the optical density change was kinetically monitored at 515 nm. The enzyme activity was expressed in units/mg protein using the catalase enzyme as the standard.

The glutathione peroxidase (GPx) activity was assayed as reported previously [12, 13, 15]. Briefly, 50 *μ*L of a sample was added to the reaction buffer consisting of 10 mM NaN_3_, 4 mM GSH, and 2.5 mM H_2_O_2_ prepared in phosphate buffer pH 7.4 and incubated for 10 min at room temperature. The GPx reaction was stopped by adding 10% trichloroacetic acid, and the unreacted GSH was estimated using 0.04% Ellman's reagent prepared in 1% sodium citrate and reading the absorbance at 412 nm using a spectrophotometer.

The glutathione reductase (GR) activity was assayed as per the method described previously [12, 13, 15]. Briefly, 50 *μ*L of a protein sample was added to the reaction mixture (0.25 M sodium phosphate buffer, pH 7.4, 0.5 mM EDTA, 4 mM oxidized glutathione, and 0.2 mM NADPH) and the oxidation of NADPH was monitored at 340 nm for 5 min. The values were expressed as units/mg of protein using GR as a standard [12, 13, 15].

### 2.6. Measurement of Energy Metabolism from Electron Transport Chain Enzyme Activity of Cardiac Mitochondria

The electron transport chain (ETC) enzyme activities were measured in mitochondria based on specific donor-acceptor oxidoreductase activities. Complexes I and III were assessed based on the rotenone-sensitive NADH-oxidoreductase (NQR) and rotenone-sensitive NADH-cytochrome C reductase (NCCR) activities. The ubiquinol cytochrome C reductase (QCR) activity was assessed for complex III, while the succinate decylubiquinone 2,6-dichlorophenolindophenol (DCPIP) reductase (SQR) and succinate cytochrome C reductase (SCCR) activities were measured for complexes II and III. The cytochrome C oxidase (COX) activity was measured as discribed [15]. All kinetic readings were acquired in a high throughput format using a using Synergy H1 multimode reader (BioTek, USA). The enzyme activities were expressed in *μ*M of NADH oxidized/min/mg protein (NQR), *μ*M of cytochrome C reduced/min/mg protein (NCCR, SCCR, and QCR), *μ*M of DCPIP reduced/min/mg protein (SQR), and *μ*M of cytochrome C oxidized/min/mg protein (COX) [15].

### 2.7. Cardiac Mitochondria Swelling Assay

The mitochondrial swelling assay was performed to assess the functional efficiency of the permeability transition pore in regulation of calcium overload induced by IRI in the myocardium. In brief, the assay was performed by incubating 150 *μ*g of mitochondrial protein in swelling buffer (120 mM KCl, 10 mM Tris-HCl, and 5 mM KH_2_PO_4_, pH 7.4) and monitoring the rate of change in absorbance at 540 nm after addition of 250 *μ*M CaCl_2_ for 20 min, according to the procedure of Martens et al. [20]. The swelling activity was reported as light scattering/mg protein at 540 nm [15].

### 2.8. Determination of Mitochondrial Membrane Potential

The mitochondrial membrane potential, an indicator of the inner and outer membrane integrity, was estimated using the rhodamine 123 (RH123) membrane-sensitive dye and calculated using the Nernst equation according to the method described by Scaduto and Grotyohann [21]. Briefly, 150 *μ*g of protein was incubated with 50 nm RH123 for 30 min at 37°C. After the incubation, the sample fluorescence outside and inside of the mitochondria was estimated at ex/em = 485/538 nm, respectively, and the membrane potential (Δ*ψ*) was represented in millivolts [15].

### 2.9. Determination of Mitochondrial Superoxide (O_2_^•−^) Generation

Dihydroethidium [or hydroethidine (DHE)] is an ethidium-based, redox-sensitive fluorescent probe, shown to be oxidized by O_2_^•−^ to form 2-hydroxyethidium (2-OH-E^+^). The assay was performed using the protocol previously adapted from Back et al. [22], and the fluorescence was measured at an excitation wavelength of 500–530 nm and an emission wavelength of 590–620 nm using a fluorimeter (Tecan, Switzerland).

### 2.10. Determination of Apoptosis Markers

Chromatin fragmentation (DNA damage), poly (ADP-ribose) polymerase (PARP) activity, and caspase 3 activity in myocardial tissues were determined as described previously [23, 24].

### 2.11. Examination of Myocardial Ultrastructure

The ventricle section from each tissue was collected after reperfusion for ultrastructural imaging, using a transmission electron microscope (JEM 1400-JEOL, MA, USA). Briefly, tissues were fixed in 4% glutaraldehyde followed by 0.1% OsO_4_ at 8°C. After dehydration of the sections with graded series of acetone and propylene oxide, the samples were fixed in an epoxy resin to obtain ultrathin sections using an ultra microtome (Ultracut R, Leica GmbH, Wetzlar, Germany). The ultracut sections were stained with uranyl acetate, followed by lead citrate, and imaged on copper grids by applying 80 kV [13].

### 2.12. Determination of GSK3*β* Activity

The GSK3*β* activity in myocardial tissues was determined using an ADP-Glo kinase assay kit (Promega, Madison, WI, USA). In brief, this luminescent kinase assay measures ADP formed in a kinase reaction, which is converted to ATP and then to luminescence with the Ultra-Glo luciferase. Luminescence was measured in a multimode plate reader (Tecan) and was directly proportional to the GSK3*β* activity present in the samples.

### 2.13. mRNA Expression of Mitochondrial Biogenesis Markers

Total RNA was extracted using the TRIzol reagent (Thermo Fisher Scientific, MA, USA) according to the manufacturer's protocol. Next, reverse transcription was performed using Verso cDNA synthesis kit (Thermo Fisher Scientific). Real-time PCR reactions were carried out using the DyNAmo Flash SYBR Green qPCR kit (Thermo Fisher Scientific) on a PCR system (ABI 7500, Applied Biosystems, Foster City, CA, USA). The amplification conditions were as follows: initial denaturation at 95°C for 2 min, followed by 35 cycles at 95°C for 30 sec and 60°C for 30 sec. The primers used in this study are listed below:

1. Nuclear respiratory factor 1 (*NRF-1*)
  - Sense (5′-GAGTGACCCAAACCGAACA-3′)
  - Antisense (5′-GGAGTTGAGTATGTCCGAGT-3′)
2. Nuclear respiratory factor 1 (*NRF-2*)
  - Sense (5′-GCAGGCCAAGATGACGAAGT-3′)
  - Antisense (5′-ACTTACACCGGCTCGGAGAA-3′)
3. Peroxisome proliferator-activated receptor gamma coactivator 1-alpha (*PGC-1α*)
  - Sense (5′-GACCACAAACGATGACCCTC-3′)
  - Antisense (5′TGTTGCGACTGCGGTTGT3′)
4. Mitochondrial transcription factor A (*TFAM*)
  - Sense (5′-GGTGTATGAAGCGGATTT-3′)
  - Antisense (5′-CTTTCTTCTTTAGGCGTTT-3′)
5. GAPDH
  - Sense (5′-GGAAGGACTCATGACCACAGT-3′)
  - Antisense (5′-GCCATCACGCCACAGTTTC-3′)

### 2.14. Molecular Docking Studies for Determining a Potent Inhibitor of GSK3*β* Activity

#### 2.14.1. Homology Modelling

Since the experimentally determined structure of GSK3*α* was not available, we modeled the protein structure using comparative modeling. The amino acid sequence of GSK3*α* with the accession number NP_063937 was downloaded from NCBI protein database. The structure of GSK3*α* was modeled using the PRIME module implemented in the Schrodinger Software Suite (Prime, Schrödinger, LLC, New York, NY, 2015). The crystal structure of GSK3*β* (PDB: 1Q41) with 2.1 A° resolution was downloaded from the RCSB Protein Data Bank (PDB) and used as a template. The quality of the model was evaluated using the discrete optimized protein energy (DOPE) statistical method for assessing homology mapping [25], the Ramachandran plot, Verify3D plot [26], and the ERRAT (program for verifying protein structures determined by crystallography).

#### 2.14.2. Protein Structure Preparation

The atomic coordinates of GSK3*β* complexed with indirubin-3′-monoxime (1Q41) were downloaded from the RCSB Protein Data Bank (PDB) [27]. The downloaded structure was prepared using the protein preparation wizard [28], a tool from the Maestro software package (Maestro v9.3, Schrödinger, LLC, New York, NY). Hetero groups were assigned appropriate bond orders. The formal charges were added, and the valences of all atoms in the structure were satisfied with hydrogens. Hydrogen bond network optimization was done by prediction of His tautomers and ionization states, assignment of 1800 rotations of the terminal *χ* angle to Asn, Gln, and His residues, and sampling of hydroxyl and thiol hydrogens. The cocrystallized ligand and crystallographic water molecules in the structure were removed. Using the OPLS-2005 force field and restrained minimization protocol, the energy of the protein was minimized with the default constraint of 0.30 A° root-mean-square deviation (RMSD). The same protocol was used for preparing the GSK3*α* model.

#### 2.14.3. Ligand Docking/Refinement

The structure of fisetin was drawn using MarvinSketch chemical structure drawing software. The energy-minimized 3D structures of fisetin were generated using the LigPrep wizard in the Schrödinger software Suite. All possible ionization states of fisetin were generated in the pH range of 5–9.

#### 2.14.4. Receptor Grid Generation

The receptor grid was generated using the Schrödinger receptor grid generation wizard. The grid box was kept centered on the cocrystallized ligand, indirubin-3′-monoxime. The size of the grid box was defined so that it covered the entire ATP-binding site.

#### 2.14.5. Molecular Docking

Molecular docking of fisetin with GSK3*α* and GSK3*β* was performed using the Schrödinger Glide extra precision (XP) algorithm (Schrödinger, LLC) [29]. The default parameters were used, and no constraints were set for the ligand-receptor interactions. The docking results were written as a pose viewer file, and the protein-ligand complex interactions were studied using the PyMOL molecular graphics system, version 1.8 (Schrödinger, LLC.).

### 2.15. Statistical Analysis

Values presented are mean ± SEM. Statistical fitness of the data was analyzed using the GraphPad Prism 7.0 program (GraphPad Software, La Jolla, CA, USA). One-way analysis of variance (ANOVA) was used to determine significant differences between the groups, and post hoc Dunnet's test was employed to determine the difference among the groups, *P* < 0.05 was considered statistically significant.

## 3. Results

### 3.1. Fisetin Attenuates I/R-Induced Myocardial Tissue Injury

**Figure 1:**
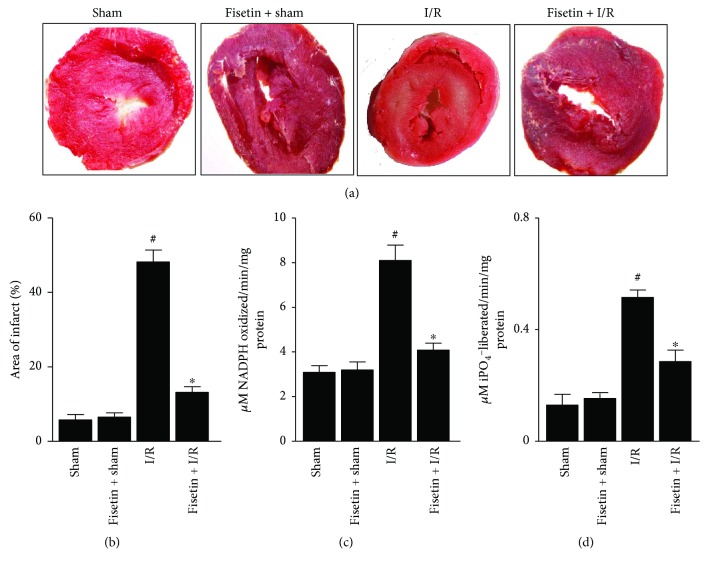
Depicts the effect of fisetin treatment on IRI. (a) Shown are the representative TTC stained sections of heart tissues from the respective groups; (b) the LDH activity in the perfusate and (d) CK activity in the perfusate. *n* = 6/group; ^#^*P* < 0.001 versus sham/sham ± fisetin; ^∗^*P* < 0.01 versus I/R.

As shown in (Figures 1(a) and 1(b)), TTC staining revealed a marked increase in the infarct size in the animals subjected to I/R. However, this effect was attenuated when animals were pretreated with fisetin. Further, analysis of LDH (Figure 1(c)) and CK (Figure 1(d)) activities in the perfusate revealed similar trends, and the I/R effects were ameliorated by fisetin treatment. Similarly, the animals subjected to I/R exhibited decreases in the indices of hemodynamic parameters (Table 1) but showed improvement in cardiac function upon treatment with fisetin (Table 1).

### 3.2. Fisetin Mitigates Oxidative Stress in Mitochondria, Lysosomes, and Microsomes

**Figure 2:**
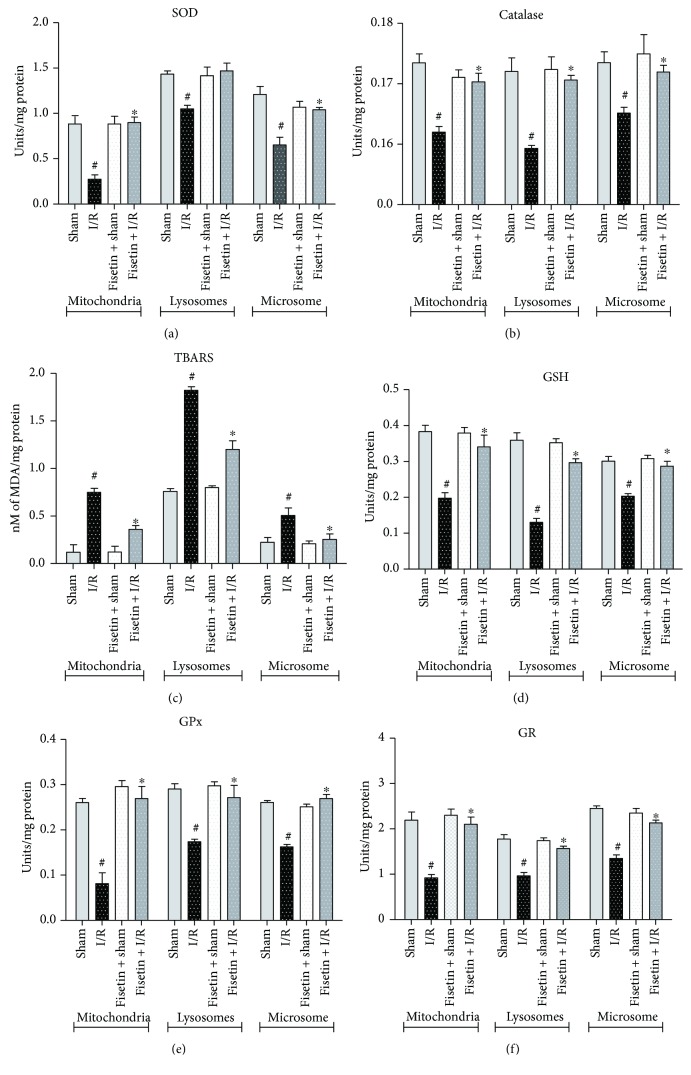
Effect of fisetin on subcellular antioxidant defense system isolated from heart tissues. (a) SOD activity, (b) catalase activity, (c) lipid peroxide accumulation, (d) total glutathione content, (e) GPx activity, and (f) GR activity in the respective groups. *n* = 6/group; ^#^*P* < 0.001 versus sham/sham ± fisetin; ^∗^*P* < 0.01 versus I/R.

In I/R-injured cardiac tissue, subcellular organelles undergo stress caused by the release of ROS from mitochondria. As mitochondria plays a major role in the I/R pathology, release of ROS can impair autophagy by affecting the structure and function of lysosomes [30]. The endoplasmic reticulum which forms the major component of microsomes is equally affected by I/R due to accumulation of unfolded proteins leading to loss of Ca^2+^ homeostasis in the myocardium. Hence, the restoration of autophagy and Ca^2+^ homeostasis and the reduction of ROS in the subcellular compartments are vital for the restoration of the normal cardiomyocyte function [30]. Therefore, we determined the oxidative stress markers such as the accumulation of lipid peroxides and endogenous antioxidants. As shown in Figure 2, the SOD activity was markedly reduced in all the subcellular compartments in myocardial tissues from the animals subjected to I/R. Likewise, the catalase (Figure 2(b)), GPx (Figure 2(e)), and GR (Figure 2(f)) activities were diminished in the animals subjected to the I/R procedure. The decrease in the endogenous antioxidant defense enzyme system corroborated a diminished GSH content (Figure 2(d)) and elevated accumulation of lipid peroxides (Figure 2(c)). However, fisetin treatment abrogated the oxidative stress and augmented the endogenous antioxidant defense system.

### 3.3. Fisetin Attenuates I/R-Induced Disruption of Mitochondrial ETC

**Figure 3:**
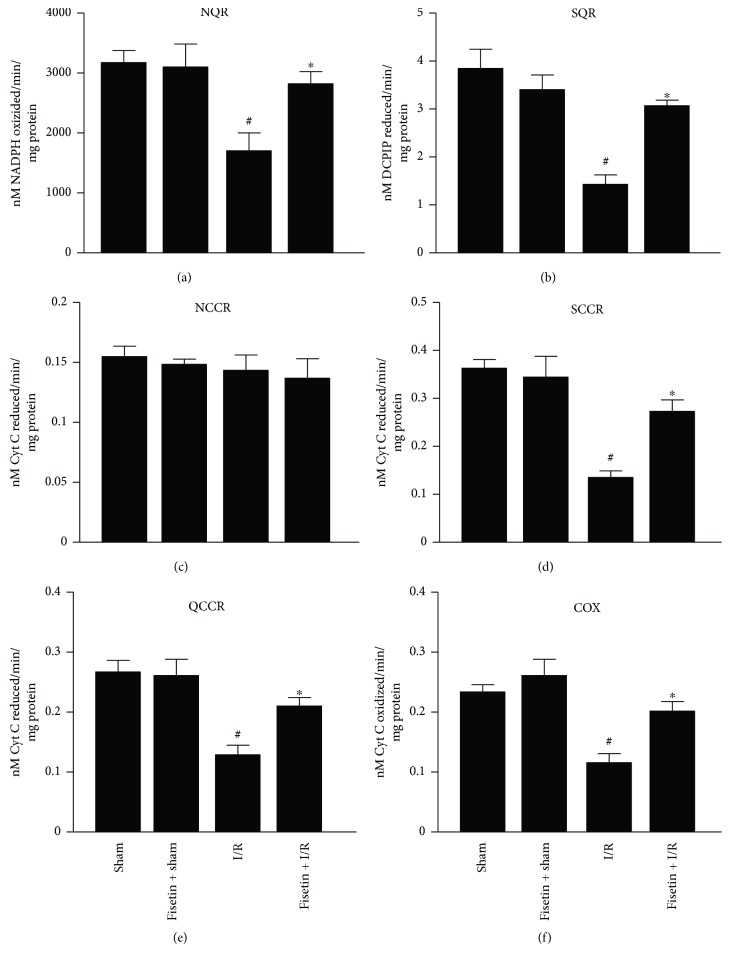
Effect of fisetin on mitochondrial enzyme activities. (a) NQR, (b) SQR, (c) NCCR, (d) SCCR, (e) QCCR, and (f) COX activities in the mitochondrial samples isolated from heart tissues. *n* = 6/group; ^#^*P* < 0.001 versus sham/sham ± fisetin; ^∗^*P* < 0.01 versus I/R.

The activities of NQR (Figure 3(a)), SQR (Figure 3(b)), SCCR (Figure 3(d)), QCCR (Figure 3(e)), and COX (Figure 3(f)) were markedly reduced in the mitochondria isolated from cardiac tissues obtained from the animals subjected to I/R. However, the NCCR activity was not affected by I/R. Moreover, fisetin treatment prevented the I/R-induced deterioration of ETC (Figure 3).

### 3.4. Fisetin Improves Mitochondrial Physiology

**Figure 4:**
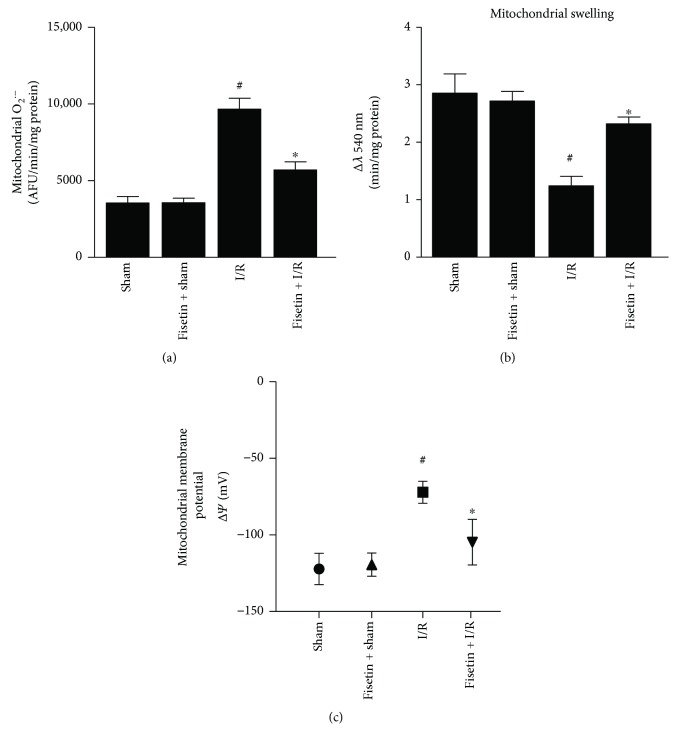
Effect of fisetin on mitochondrial physiology. (a) The mitochondrial superoxide generation, *n* = 6/group; ^#^*P* < 0.001 versus sham/sham ± fisetin; ^∗^*P* < 0.01 versus I/R. (b) Mitochondrial swelling activity, *n* = 6/group; ^#^*P* < 0.001 versus sham/sham ± fisetin; ^∗^*P* < 0.01 versus I/R. (c) Mitochondrial membrane potential, *n* = 6/group; ^#^*P* < 0.01 versus sham/sham ± fisetin; ^∗^*P* < 0.05 versus I/R.

There was a marked generation of mitochondrial O_2_^•−^ in the myocardial tissues obtained from the animals subjected to I/R (Figure 4(a)). Similarly, mitochondrial swelling increased (Figure 4(b)) and the membrane potential was lost in the I/R subjected animals (Figure 4(c)). However, these effects were attenuated by fisetin treatment.

### 3.5. Fisetin Mitigates I/R-Induced Apoptosis

**Figure 5:**
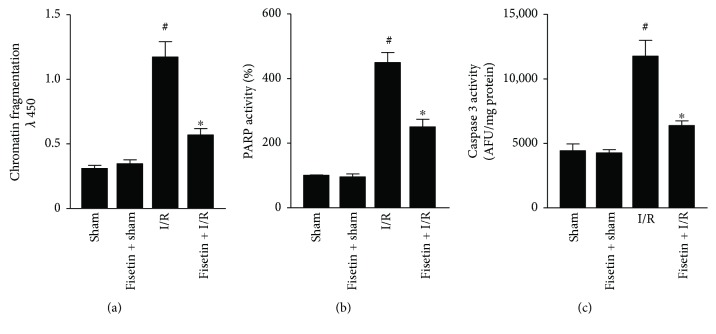
Effect of fisetin on myocardial apoptosis induced by IRI. (a) The DNA fragmentation, (b) PARP, and (c) caspase 3 activities in the heart tissues. *n* = 6/group; ^#^*P* < 0.001 versus sham/sham ± fisetin; ^∗^*P* < 0.01 versus I/R.

Loss of the mitochondrial membrane potential triggers the apoptosis cascade, which eventually results in the demise of cardiomyocytes. We observed a greater propensity for apoptosis in the I/R group, which was characterized by increased DNA fragmentation (Figure 5(a)) and elevated PARP and caspase 3 activities (Figures 5(b) and 5(c)). However, fisetin treatment blunted the I/R-induced apoptotic response in myocardial tissues.

### 3.6. Fisetin Augments Mitochondrial Biogenesis

**Figure 6:**
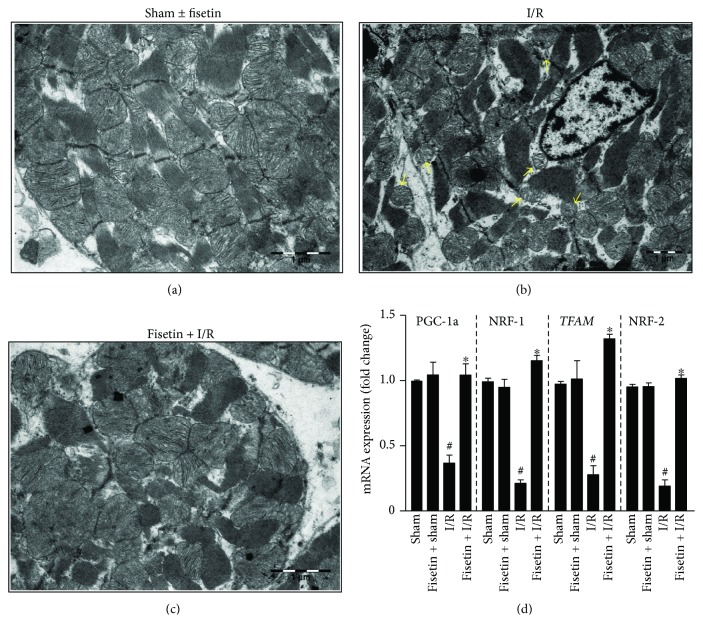
Effect of fisetin on myocardial tissue ultrastructure examined by electron microscope (EM) and mitochondrial biogenesis makers determined by qRT-PCR technique. (a) Representative EM picture of myocardial tissue processed from sham or sham ± fisetin-treated animals. (b) Shown is the EM image of myocardial tissue from animals subjected to IRI; yellow arrowheads indicate that mitochondria are markedly abnormal, with varying size and densities and characterized by the loss of matrix and cristae. (c) EM picture reveals that fisetin attenuates mitochondrial damage induced by IRI. (d) The mRNA expression of mitochondria biogenesis markers; *n* = 6/group; ^#^*P* < 0.001 versus sham/sham ± fisetin; ^∗^*P* < 0.01 versus I/R.

First, we determined the impact of I/R on the mitochondrial histoarchitecture with the aid of transmission electron microscope. We observed that the induction of IRI resulted in interspersed vacuoles and a disarray of the myofibrillar lattice. Furthermore, mitochondria were markedly abnormal, with varying sizes and densities and characterized by a loss of the matrix and cristae (Figure 6(b)). However, fisetin treatment improved the mitochondrial histoarchitecture (Figure 6(c)). Next, we determined the mRNA expression of key meditators involved in the mitochondrial biogenesis. We observed that the gene expression of *PGC1-α*, *NRF-1*, NRF-2, and *TFAM* was significantly diminished in myocardial tissues obtained from the animals subjected to the I/R procedure. However, fisetin treatment augmented the mitochondrial biogenesis, which was evident from the reversal of the mRNA expression levels of *PGC1-α*, *NRF-1*, and *TFAM* (Figure 6(d)).

### 3.7. Fisetin Potently Inhibits GSK3*β* Activity

**Figure 7:**
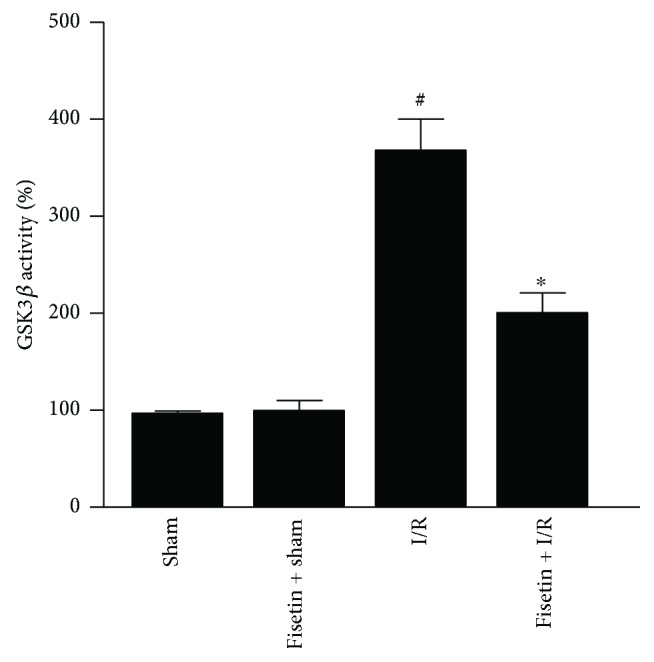
Fisetin inhibits GSK3*β* activity in myocardial IRI tissues; *n* = 6/group; ^#^*P* < 0.001 versus sham/sham ± fisetin; ^∗^*P* < 0.01 versus I/R.

GSK3*β* activation has been reported to be associated with the defective mitochondrial physiology during cardiac I/R [31]. Therefore, there is immense interest in developing potent and selective inhibitors of GSK3*β*, which could be used in the clinic to minimize IRI. Accordingly, we determined whether fisetin could inhibit the GSK3*β* activity in the myocardial tissues obtained from the I/R-subjected animals. The results showed that GSK3*β* was elevated in I/R tissues but this increase was inhibited by fisetin (Figure 7). Based on this biochemical data, we performed a detailed bioinformatics analysis to characterize fisetin as a potent and selective inhibitor of GSK3*β*.

### 3.8. In Silico Analysis to Characterize Fisetin as an Inhibitor of GSK3*β*

**Figure 8:**
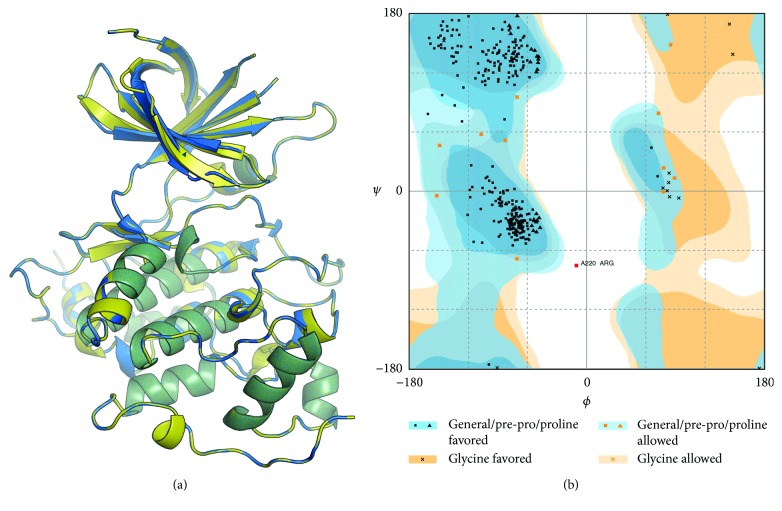
Comparative modeling of GSK3*α* using the crystal structure of GSK3*β* (PDB: 1Q41). (a) Molecular superimposition of GSK3*α* and GSK3*β*. Crystal structure of GSK3*β* and modeled GSK3*α* were shown in blue and yellow. (b) Ramachandran plot of the modeled GSK3*α*.

**Figure 9:**
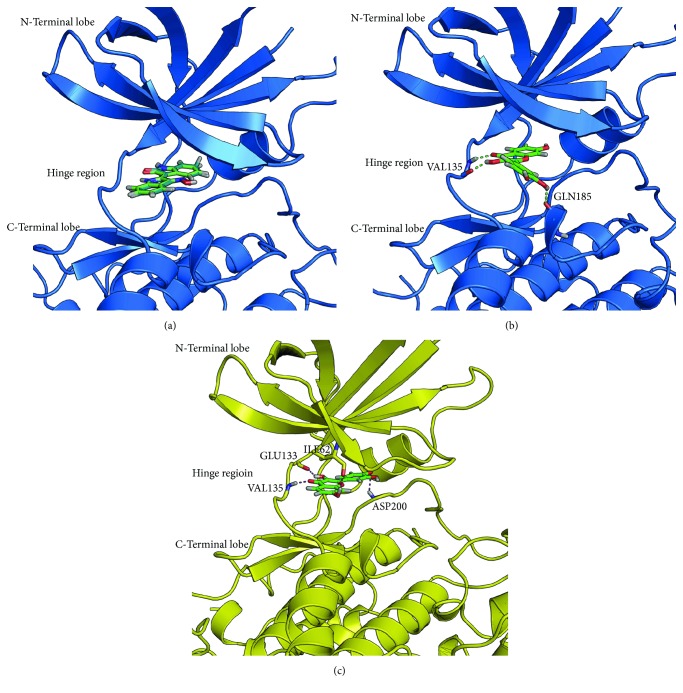
Molecular docking and hydrogen bonding interactions. (a) Molecular redocking of indirubin-3′-monoxime with GSK3*β*. The crystal and redocked poses are shown in green and blue, respectively. (b) Molecular docking and hydrogen bonding interactions of fisetin with GSK3*α*. Hydrogen bonding interaction is shown as dotted green lines. (c) Molecular docking and hydrogen bond interactions of fisetin with GSK3*β*. Hydrogen bond interactions are shown as dotted red lines.

We used the crystal structure of GSK3*β* (1Q41) as a template for modeling the GSK3*α* structure. The sequence identity between GSK3*α* and GSK3*β* is 77%. The missing loops were modeled and refined. The modeled protein was subjected to a brief constrained energy minimization, and the calculated potential energy (OPLS3) of the modeled protein (GSK3*α*) was 10,513 kcal/mol. The calculated RMSD between the modeled GSK3*α* and the GSK3*β* template was 0.92 A° (Figure 8(a)). The stereochemical quality of the model was evaluated using the Ramachandran plot (Figure 8(b)). Approximately 96.4%, 3.3%, and 0.3% of the amino acids were present in the most favored, allowed, and outlier regions, respectively. The ERRAT value for all quality factors of the protein model was 88.21%. The Verify3D results showed that 89.09% of the residues had an average 3D-1D score of ≥0.2 in the 3D/1D profile. To evaluate the efficiency of the docking algorithm to reproduce the crystal pose, we removed indirubin-3′-monoxime and redocked it using the Glide XP docking algorithm. The Glide docking algorithm was superior in reproducing the crystal pose as shown in (Figure 9(a)). Further Glide was used to dock fisetin into the binding site of GSK3*β* and GSK3*α*. The Glide docking score for the GSK3*β*-fisetin complex was −10.067 and that for the GSK3*α*-fisetin complex was −6.40. Glide score shows that fisetin has a higher affinity for the GSK3*β* than the GSK3*α*.

The docking analysis of the GSK3*β*-fisetin complex revealed that fisetin formed three strong hydrogen bond interactions with the backbone of the amino acids Val135 and Gln185 (Figure 9(b)). In the case of GSK3*α*, fisetin was able to form hydrogen bond interaction with Ile 62, Glu133, Val135, and Asp200 (Figure 9(c)). The hydrogen bond interaction patterns observed in our docking study were consistent with the interactions observed in the crystal structures of various known GSK3*β*-inhibitor complexes (PDB: 1PYX, 5HLN, 5HLP, 1Q41, and 3SAY) [32, 33]. Further analysis showed that the preferred orientations for fisetin binding to GSK3*α* and GSK3*β* were completely different (Figures 9(b) and 9(c)). The docked pose of fisetin with GSK3*α* showed that all the ring systems of the fisetin molecule were in the same plane and the dihydroxy phenyl group was projected away from the hinge region. However, with GSK3*β*, the dihydroxy phenyl group of fisetin was slightly projected out of the plan and closer to the hinge region, and this conformation leads to the formation of hydrogen bonding with Gln185. This shows that the orientation of the dihydroxy phenyl ring of fisetin and its interactions with Gln185 could play a role in its affinity and selectivity. These analyses led to the conclusion that fisetin could be a potent and isoform-specific GSK3*β* inhibitor.

## 4. Discussion

Mitochondria are recognized as the primary target of the IRI in the myocardium. Timely reperfusion is performed to salvage the healthy myocardium after ischemic injury [34]. Efforts to unravel the potential pathomechanisms of IRI are restrained because of the lack of a bonafide biomarker and definitive clinical endpoints. During IRI, the damage to the mitochondrial ETC chiefly occurs during the ischemic phase [35]. The reperfusion phase exacerbates the injury in the myocardium, which occurs during the previous phase of ischemic insult, and this drives an excessive ROS production and perturbation of Ca^2+^ homeostasis, resulting in abnormal mitochondrial membrane permeability and increased swelling [36]. Furthermore, increased mitochondrial ROS production can result in decreased oxidative phosphorylation and can jeopardize the functional recovery of the ailing myocardium after IRI. It is pertinent to note that the adequate supply of ATP is crucial for augmenting the metabolic activity, aiding in collateral circulation (neoangiogenesis), which is of paramount significance for improving the cardiac function [37].

In the present study, we observed significant myocardial tissue injury in animals subjected to myocardial IRI, as revealed by compromised cardiac function. However, when animals were pretreated with fisetin, there was a marked improvement in cardiac function and decrease of myocyte injury markers such as LDH and CK. Prior studies have reported that fisetin showed antioxidant activity and ameliorated various inflammatory diseases in preclinical studies [38–40].

Mitochondrial permeability transition pore (mPTP) opening results in destabilization of mitochondrial membrane integrity, and this leads to the extrusion of ionic contents [41]. Maintenance of mitochondria health during I/R plays a key role in the recovery of myocardium. Especially, the opening of mPTP during early stage of reperfusion injury is shown to increase the myocardial infarct size and the drugs inhibiting mPTP opening can provide cardioprotection by preserving mitochondrial structure and function [42]. mPTP is shown to mediate the apoptosis by rendering the pore permeable to molecule release from the matrix to the cytosol, thereby disrupting the membrane potential and uncoupling the oxidative phosphorylation. Therefore, to assess the effect of fisetin on the mPTP opening, we measured the Ca^2+^-induced swelling behavior due to the I/R in the mitochondria isolated from the heart tissues. Our results indicated impaired swelling behavior due to I/R which was improved in samples obtained from fisetin-pretreated animals. In addition, we observed that membrane potential (Δ*ψ* mV) in fisetin + I/R group remained hyperpolarized, compared to I/R group. These observations suggest that fisetin inhibits mPTP opening and improves the mitochondrial function, thereby preventing I/R-induced myocardial tissue injury. Our findings are in agreement with a previous study, which demonstrated that fisetin could counteract oxidative stress-induced renal tissue damage by improving the oxidative phosphorylation and improving the mitochondrial complex activities [43].

Because of the restricted rate of cardiomyocyte renewal following cardiomyocyte death, in both the injured and adjacent area upon IRI, the adult heart is unable to restore the damaged tissue upon myocardial infarction [44]. Instead, scar formation is induced in the damaged zone resulting in a loss of contractility rather than in myocardial tissue repair. Moreover, the myocardial tissue ceaselessly functions to provide ATP and regulate the metabolic tone of the heart, which has one of the highest oxygen consumption rates in the body, mostly through aerobic metabolism [45]. Since cardiomyocytes are terminally differentiated, they cannot be easily replenished. Therefore, cardiomyocytes are sensitive to any abnormal stress or stimuli, and consequently, an efficient antioxidant defense system is vital for the prevention IRI-induced damage to cardiomyocytes. During IRI, ROS accumulation over time overpowers the antioxidant capacity of mitochondria, which is primarily provided by SOD, catalase, GPx, and GR. When the antioxidant rheostat malfunctions during IRI, this culminates in mitochondrial dysfunction and subsequently affects the cardiac function [4]. In this study, we observed that fisetin treatment could potentially save the mitochondria from IRI by bolstering their antioxidant capacity.

Cardiac myocytes undergo profound age-related alterations which are necessary for the sustenance and survival during the lifespan of an individual. Autophagy (self-cannibalism) is a mechanism employed by cardiomyocytes for their survival under physiological and pathological conditions [30]. It has been reported that the accumulation of defective autophagosomes are associated with the pathogenesis of several cardiovascular diseases, including IRI [30]. Defective autophagosomes can contain damaged mitochondria, unfolded protein aggregates, and other globular complex lipid-protein complexes [30]. Under physiological conditions, cardiomyocyte lysosomes contain digestive enzymes such as calpain, which process these cellular waste products [46]. However, during I/R, the mitochondria swell due to dysfunctional mPTP that leads to recruitment of Parkin, which activates calpain-1 and promotes autophagy. Furthermore, these events are fueled by vicious cycle of increased ROS generation that culminates in the heart failure [46, 47]. Similarly, a premature rupture of lysosomes releases a labile iron pool from hydrolytic enzymes, which perpetuates Fenton's reaction and propagation of oxidative stress, which in turn inflicts lysosomal injury through oxidative modification of lipids and proteins [48].

Therefore, stable lysosomes are vital for the sustenance of healthy cardiomyocytes and their protection from deleterious effects of IRI [49]. In this regard, the effective antioxidant capacity is vital for the prevention of lysosome destabilization during IRI [49]. In our present study, we observed that lysosomes and microsomes obtained from IRI-affected hearts exhibited exaggerated oxidative stress, characterized by diminished levels of endogenous antioxidants and accumulation of lipid peroxides. However, treatment with fisetin blunted the oxidative stress and this effect corroborated with the improved cardiac function. Additionally, previous studies have reported that fisetin significantly attenuated rotenone-induced neuroinflammation, by scavenging ROS at mitochondria. In fact, previous studies have documented that fisetin could engage NRF-2 and augment the expression of hemooxugenase-1 (HO-1) as well as confer protection against oxidative insult in human endothelial cells [50].

Apoptotic cell death is the key feature of I/R-induced myocardial tissue injury and heart failure. Previous studies have reported that the treatment with natural or synthetic antioxidants could be beneficial in thwarting the IRI-induced myocardial tissue injury [5]. Herein, we have observed that IRI-affected heart tissues exhibited increased DNA fragmentation, which was accompanied by increased PARP and caspase 3 activities, signifying an increased rate of apoptosis. Remarkably, fisetin treatment blunted the apoptotic response in agreement with a previous report showing that fisetin elicited an antiapoptotic effect during inflammatory stress [51].

Mitochondrial biogenesis is a process of the replenishment of damaged/defective mitochondria in cells, and this phenomenon is universally observed in most cells and tissues [52]. It is pertinent to note that defective mitochondrial biogenesis has been reported in myocardial IRI and other cardiovascular diseases [53]. Therefore, defective mitochondrial biogenesis in cardiomyocytes during IRI could hamper myocardial recovery and eventually result in heart failure [53]. In our present study, we observed that mRNA expression of key transcription factors regulating mitochondrial biogenesis such as *PGC-1α*, *NRF-1*, and *TFAM* was reduced in the hearts obtained from IRI animals. However, mitochondrial biogenesis improved when the animals were treated with fisetin. Previous studies have reported that fisetin could augment the mitochondrial biogenesis in adipocytes via PGC-1*α* activation, and our present findings are consistent with these observations [54].

GSK3*β* is a serine/threonine kinase playing a pivotal role during development, cell proliferation, migration, differentiation, modulation of apoptosis, and oncogenesis [55]. Previous studies have indicated that inhibition of the GSK3*β* activity could confer cardioprotection against IRI [9]. GSK3*β* has been reported to regulate PGC-1*α* degradation. GSK3*β* represses the PGC-1*α* activation, through phosphorylation and thus priming PGC-1*α* for degradation via the ubiquitin-proteasome pathway [56]. Inhibition of GSK3*β* promotes the mitochondrial biogenesis during ischemia cerebral injury [57]. Moreover, defective GSK3*β* activity is linked to dysregulated mitochondrial biogenesis [56]. Therefore, we determined the GSK3*β* activity in myocardial tissues obtained from respective groups of mice and found that GSK3*β* activity was significantly increased in the samples from IRI group, but the increase was suppressed by fisetin treatment. Moreover, it is pertinent to note that fisetin has been reported to attenuate the GSK3*β* activity *in vivo* during neuroinflammatory insult in preclinical studies [58]. Therefore, it is likely that fisetin could recruit PGC-1*α*, augment the mitochondrial biogenesis, repress the mitochondrial oxidative stress, and ameliorate the myocardial IRI. To further confirm our *in vivo* findings, in silico molecular docking studies were performed to ascertain whether fisetin could serve as a selective and potent inhibitor of GSK3*β* activity. Our results suggested that the compound could selectively inhibit the GSK3*β* activity.

**Figure 10:**
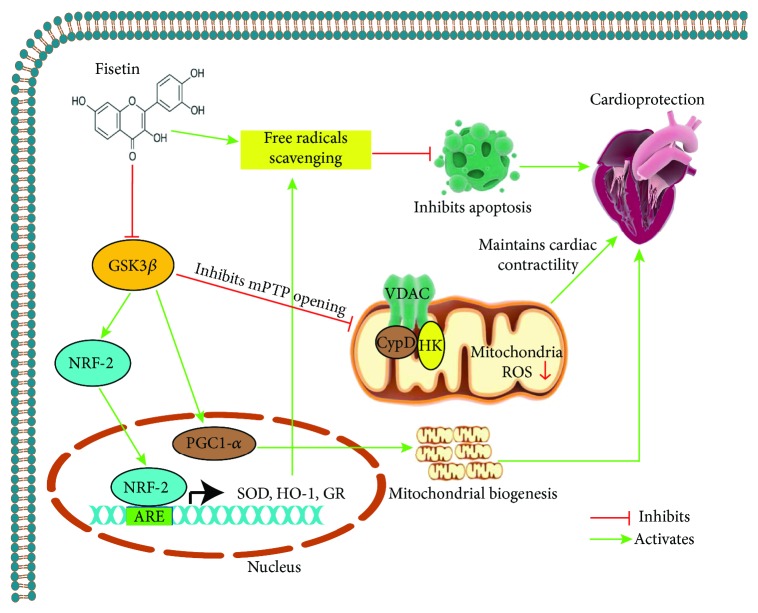
This scheme shows the fisetin cardioprotective effects against myocardial tissue injury upon I/R. Fisetin scavenges ROS and prevents mitochondrial dysfunction by inducing the Nrf-1/2 expression, bolstering the mitochondrial physiology, and inhibiting the GSK3*β* activity.

In summary, our definitive and novel findings obtained in this study indicate that fisetin confers cardioprotection against myocardial IRI, by bolstering the mitochondrial physiology, suppressing the oxidative stress, and augmenting the mitochondrial biogenesis, and these effects are mediated via inhibition of GSK3*β* activity (Figure 10). Since fisetin is well tolerated in human subjects and does not show toxic effects, this natural small molecule has bright prospects for further pharmaceutical development to be used against I/R-induced myocardial tissue injury and potentially for the treatment of cardiovascular diseases.

## Figures and Tables

**Table 1:** Effect of fisetin on hemodynamic parameters.

Group	LVSP	LVDP	RPP	+dp/dt	−dp/dt
	(mmHg)	(mmHg)	(mmHg^∗^ BPM × 10^3^)	(mmHg/s)	(mmHg/s)
Sham (*n* = 8)	120.0 ± 2.2	13 ± 1.3	36.3 ± 0.26	3001.0 ± 23.3	3207.0 ± 13.4
Fisetin + sham (*n* = 8)	117.0 ± 5.2	12 ± 2.2	35.7 ± 0.23	2994.0 ± 21.2	3200.0 ± 14.2
I/R (*n* = 8)	27.0 ± 2.9^∗^	16 ± 3.1^∗^	4.7 ± 0.34^∗^	1729.7 ± 22.6^∗^	1126.7 ± 12.3^∗^
Fisetin + I/R (*n* = 8)	110.8 ± 2.5^#^	13 ± 2.6^#^	29.3 ± 0.25^#^	2842.0 ± 26.9^#^	2974.3 ± 15.5^#^

LVSP: left ventricular systolic pressure; LVDP: left ventricular diastolic pressure; RPP: rate pressure product; ±dp/dt: ventricular contraction assessment. ^∗^*P* < 0.001 versus sham/fisetin + sham; ^#^*P* < 0.01 versus I/R.
